# Prospective head-to-head comparison of accuracy of two sequencing platforms for screening for fetal aneuploidy by cell-free DNA: the PEGASUS study

**DOI:** 10.1038/s41431-019-0443-0

**Published:** 2019-06-23

**Authors:** François Rousseau, Sylvie Langlois, Jo-Ann Johnson, Jean Gekas, Emmanuel Bujold, François Audibert, Mark Walker, Sylvie Giroux, André Caron, Valérie Clément, Jonatan Blais, Tina MacLeod, Richard Moore, Julie Gauthier, Loubna Jouan, Alexandre Laporte, Ousmane Diallo, Jeremy Parker, Lucas Swanson, Yongjun Zhao, Yves Labelle, Yves Giguère, Jean-Claude Forest, Julian Little, Aly Karsan, Guy Rouleau

**Affiliations:** 1 Department of Laboratory Medicine, CHU de Québec-Université Laval & Department of Molecular Biology, Medical Biochemistry and Pathology, Faculty of Medicine, Université Laval, Québec City, QC, Canada; 2 Centre de recherche du CHU de Québec–Université Laval, Québec City, QC, Canada; 3 Department of Medical Genetics, University of British Columbia, Vancouver, BC Canada; 4 Dept. of Obstetrics & Gynecology, University of Calgary, Calgary, AB Canada; 5 Department of Pediatrics, Mother and Child Center, University Hospital of Quebec, Laval University Research Center, Québec City, QC, Canada; 6 Department of Obstetrics and Gynaecology, Laval University, Québec City, QC, Canada; 7 Department of Obstetrics and Gynecology, CHU Ste-Justine Research Center, Université de Montréal, Montréal, QC Canada; 8 Department of Obstetrics & Gynecology, University of Ottawa, Ottawa, ON Canada; 9 The Ottawa Hospital Research Institute, Ottawa, ON Canada; 10 School of Epidemiology and Public Health, University of Ottawa, Ottawa, ON Canada; 11 Department of Laboratory Medicine, CISSS Chaudière-Appalaches, Lévis, QC Canada; 12 Department of Molecular Biology, Medical Biochemistry and Pathology, Faculty of Medicine, Université Laval, Québec City, QC, Canada; 13 Michael Smith Genome Sciences Centre, BC Cancer Agency, Vancouver, BC Canada; 14 Molecular Diagnostic Laboratory and Division of Medical Genetics, CHU Sainte-Justine, Montreal, QC Canada; 15 Montreal Neurological Institute, Department of Neurology and Neurosurgery, McGill University, Montreal, QC Canada; 16 Department of Pathology & Laboratory Medicine, BC Cancer Agency, Vancouver, BC Canada

**Keywords:** Laboratory techniques and procedures, Diagnostic markers

## Abstract

We compared clinical validity of two non-invasive prenatal screening (NIPS) methods for fetal trisomies 13, 18, 21, and monosomy X. We recruited prospectively 2203 women at high risk of fetal aneuploidy and 1807 at baseline risk. Three-hundred and twenty-nine euploid samples were randomly removed. The remaining 1933 high risk and 1660 baseline-risk plasma aliquots were assigned randomly between four laboratories and tested with two index NIPS tests, blind to maternal variables and pregnancy outcomes. The two index tests used massively parallel shotgun sequencing (semiconductor-based and optical-based). The reference standard for all fetuses was invasive cytogenetic analysis or clinical examination at birth and postnatal follow-up. For each chromosome of interest, chromosomal ratios were calculated (number of reads for chromosome/total number of reads). Euploid samples’ mean chromosomal ratio coefficients of variation were 0.48 (T21), 0.34 (T18), and 0.31 (T13). According to the reference standard, there were 155 cases of T21, 49 T18, 8 T13 and 22 45,X. Using a fetal fraction ≥4% to call results and a chromosomal ratio *z*-score of ≥3 to report a positive result, detection rates (DR), and false positive rates (FPR) were not statistically different between platforms: mean DR 99% (T21), 100%(T18, T13); 79%(45,X); FPR < 0.3% for T21, T18, T13, and <0.6% for 45,X. Both methods’ negative predictive values in high-risk pregnancies were >99.8%, except for 45,X(>99.6%). Threshold analysis in high-risk pregnancies with different fetal fractions and *z*-score cut-offs suggested that a *z*-score cutoff to 3.5 for positive results improved test accuracy. Both sequencing platforms showed equivalent and excellent clinical validity.

## Introduction

### Scientific and clinical background

Since the discovery that maternal blood during pregnancy carries detectable amounts of circulating cell-free DNA (ccfDNA) fragments shed from the placenta [1, 2], various methods have been proposed to identify major fetal aneuploidies by massively parallel sequencing of either selected or unselected ccfDNA [3]. Although studies of the performance of non-invasive prenatal screening (NIPS) using cfDNA in maternal blood have demonstrated a superior performance compared to standard screening based on serum biomarkers and nuchal translucency measurement, there is variation between studies in estimates of diagnostic sensitivity and specificity, as well as in test failure and false positive rates [3, 4]. Screening performances are also known to differ between trisomies 13, 18, and 21 and sex chromosome aneuploidies (SCAs) [3]. As most NIPS assays have to be developed and implemented in specific laboratories, they must be thoroughly validated by each laboratory prior to any clinical offering (International Organization for Standardization’s ISO15189).

NIPS, first implemented as a second-tier screening test for women with positive traditional screening results [5, 6], has also been proposed as a replacement for traditional fetal aneuploidy screening tests offered universally (International Society for Prenatal Diagnosis). However, there are currently less data on performance than for second-tier screening [3] and there is no consensus amongst existing guidelines on the clinical utility of NIPS as a universal screening test for fetal aneuploidy.

Best practice guidelines [7] and published Health Technology Assessments [8–10] of NIPS for fetal aneuploidy all agree that, due to the imperfect positive predictive value (PPV) of the test even in high-risk pregnancies, NIPS is not a diagnostic test and that, because of possible confined placental mosaicism, maternal mosaicism, and other biological causes for false-positive NIPS results, any positive NIPS result must be confirmed by invasive testing (either CVS or amniocentesis) with a definitive chromosomal analysis. NIPS remains a screening test designed to identify pregnancies at increased risk of common fetal aneuploidies whereas chromosome analysis of samples obtained by CVS or amniocentesis is a diagnostic test to determine, with as much certainty as possible, whether aneuploidy is present in the fetus.

Whereas Next Generation Sequencing (NGS) technologies have the same general aim of parallel sequencing millions of DNA fragments, their costs in infrastructure (equipment and computer hardware), as well as their throughput, vary widely within and between NGS vendors. Thus, depending on the expected throughput of the clinical laboratory offering NIPS services, its financial resources as well as the needed turnaround time, different NGS platforms and technologies may be considered. No study comparing head-to-head the diagnostic accuracy of NIPS based on different NGS platforms on the same patient samples has been published. Two types of NGS technologies widely used in clinical services are optical NGS (such as the Sequencing by Synthesis™ technology commercialized by Illumina) and semiconductor NGS (such as the IonTorrent™ technology commercialized by ThermoFisher) [11].

### Study objectives and hypotheses

The aim of the present study was to compare head-to-head, using the same prospective samples, the clinical performance of two massively parallel shotgun sequencing (MPSS) NIPS methods using either an optical NGS technology (Illumina) or a semiconductor NGS technology (ThermoFisher) in high risk and in baseline risk pregnancies for the detection of trisomies 21, 18, 13, and monosomy X. These specific chromosomal abnormalities were chosen as they represented the common aneuploidies currently detected by the Canadian prenatal genetic screening programs.

Our hypothesis was that, providing that the NIPS assays rely on solid clinical NGS practices and quality standards, both NGS technologies would show similar clinical performances.

## Materials and methods

### Study design

We performed a comparative diagnostic accuracy study in which we recruited prospectively pregnant women who had already been identified as either at high risk, or at baseline risk, of fetal aneuploidy. All participating women had their blood collected in large enough quantities to be tested by two NGS methods. ccfDNA grade plasma from all NIPS samples was prepared and frozen until NIPS testing. The testing was performed blindly to the reference standard after the end of pregnancy, with no communication of NIPS results. NIPS testing was performed in public laboratories that had setup their own NIPS assay. The two NGS platforms chosen for comparision (ThermoFisher’s Proton™ with IonTorrent™ sequencing and Illumina’s HiSeq2500™ with Sequencing-by-synthesis™ sequencing) use very different sequencing methods. They were the most widespread in clinical laboratories when the study was initiated. ThermoFisher’s Proton™ appeared to offer versatility in terms of number of patients per run at a lower price (especially for equipment) and a shorter sequencing turnaround time, while Illumina’s HiSeq2500™ was an established method for NIPS in laboratories with a large throughput of samples. This study was registered in clinicaltrial.gov as NCT01925742.

### Participants

We obtained informed consent and recruited prospectively 2203 women at high risk and 1807 at baseline risk. Women were recruited at the Children’s & Women’s Health Centre in Vancouver (BC), the Foothills Medical Centre in Calgary (AB), The Ottawa Hospital in Ottawa (ON), the CHU Ste-Justine in Montreal (QC), and the CHU de Québec in Québec City (QC) over a period between November 2013 and April 2016. The study was granted Research Ethics Board approval in all five recruiting centers.

Inclusion criteria for high-risk pregnancies were women 19 years or older, between 10 weeks and 23 weeks and 6 days of gestational age, who qualified for invasive testing because of an increased risk of fetal aneuploidy. Pregnant women were defined as high risk if they qualified for invasive testing based on one or more of the following criteria: abnormal ultrasound findings; previous trisomy 13, 18, or 21; patient or partner carrier of a translocation; maternal age 40 years or greater; positive screening result for Down syndrome or trisomy 18 (First Trimester Screening, Serum Integrated Prenatal Screening, Integrated Prenatal Screening, Quad screening); positive second or first tier NIPS.

For the baseline risk arm, we recruited women 19 years and older, between 10 and 13 weeks and 6 days of gestation, and who decided to undergo prenatal screening for Down syndrome (contingent First Trimester Screening, Serum Integrated Prenatal Screening or Integrated Prenatal Screening). For both groups, we excluded women with a multiple pregnancy, twin demise (spontaneous or elective), or with a history of malignancy. Each participant was given a PEGASUS_ID identification number that was used for clinical data collection as well as for sample identification. Participant identification was kept separate and inaccessible to all researchers except those who were responsible for collection of participant follow-up data.

Maternal blood samples, 20–40 mL per participant, were collected prior to any invasive procedure. Samples were collected in EDTA tubes when it was possible to transport them to one of the three NIPS laboratories (Vancouver, Montreal, or Québec City) within 8 h for plasma processing (see below) (*n* = 2174). Otherwise, samples were collected in 10 mL Cell-free DNA BCT tubes from Streck™ (La Vista, NE) (*n* = 1405). For 14 samples no information on the type of sampling tube was recorded.

Upon arrival at the NIPS laboratory, plasma was separated by two centrifugation steps (1600 × *g* for 10 min followed by 16,000 × *g* for 10 min) and stored at −80 °C between 3 and 30 months in the laboratory that had performed the plasma purification steps until the NIPS assay was performed.

Clinical and follow-up data collected included *maternal variables*: ethnicity, age, weight and height at recruitment, gravidity and parity; *pregnancy variables*: spontaneous conception or IVF, ultrasound dating information, gestational age at the time of blood draw, and results of traditional prenatal screening (all baseline risk and some high risk); and *qualifying information*: indication for high risk (high-risk group only), type of sampling tube, interval between plasma purification and NIPS assay, invasive prenatal diagnostic procedure, and gestational age at delivery.

### Test methods

#### Randomization of samples between laboratories and for order of NIPS testing

In order to minimize batch testing effects, biases or confounding variables that could affect NIPS test results, while allowing studying the variables for which we collected information, we proceeded to a double randomization of samples before they were analyzed.

**Fig. 1:**
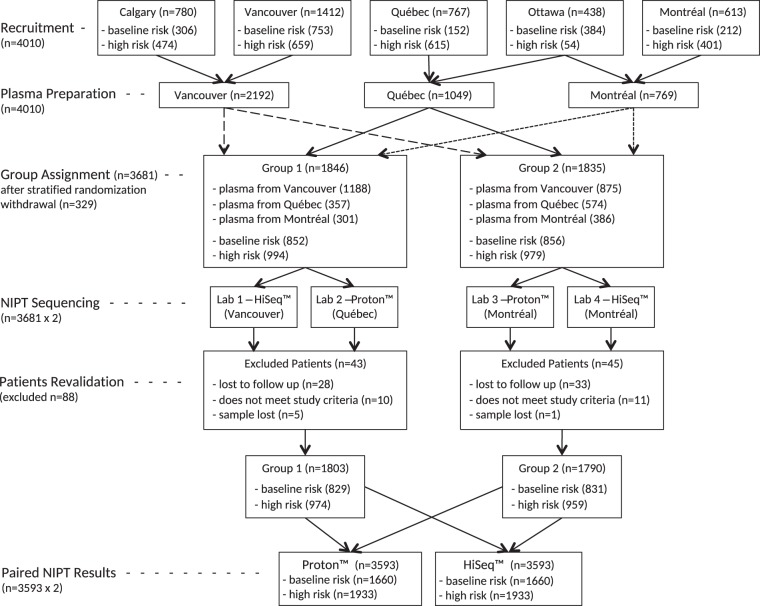
Participants flow chart

Prior to processing the plasma samples for ccfDNA purification and NIPS testing, stored samples were initially randomized between two groups of NIPS laboratories. Each group (Group 1 and Group 2; Fig. 1) had one laboratory with a HiSeq™ setup and one laboratory with a Proton™ setup. Randomization was performed centrally and blind to the study arm (high risk or baseline risk) and pregnancy outcomes. Ninety-nine baseline risk and 230 high-risk euploid samples were removed randomly prior to Group 1 vs Group 2 randomization to reduce the overall project’s NIPS testing costs while keeping sufficient statistical power for the study of test clinical specificity.

Once all samples to be tested were received by the laboratories, samples were randomized again independently (i.e. centrally and independently from the laboratories) to determine their testing order and were analyzed sequentially using this randomized order.

#### Index tests

All samples were tested blind to the reference standard, the risk level for fetal aneuploidy, the pregnancy outcomes as well as to clinical characteristics of the pregnant women. In order to detect and quantify potential cross-contamination between patient samples during the NIPS testing processes, each patient sample was spiked with different synthetic barcodes DNA into the plasma just before the cfDNA extraction and were detected and quantified during the NGS step. DNA was extracted from 5 ml of plasma using the QIAamp Circulating Nucleic Acid Kit (Qiagen, Hilden, Germany) or from 2 ml of plasma using the QIAsymphony Viral/Pathogen DSP kit (Qiagen). All samples were tested by two MPSS methods (optical-based [or HiSeq™] and semiconductor [or Proton™] sequencing). All samples were tested only once by each index test and were not re-tested nor re-sequenced if they either failed QC thresholds or if the fetal fraction estimate was below 4%.

For the optical-based sequencing platform, shotgun libraries for the Illumina platform were either constructed by using a modified version of an automated plate-based Illumina genome protocol [12] or using TruSeq nano Kit (Illumina) according to the manufacturer’s recommendations with minor modifications. An initial bead-based size selection using ALine PCRClean™ Dx magnetic beads (Aline Biosciences, USA) or Agencourt AMpure beads (Beckman Coulter, Brea,CA) at 0.8:1 ratio to enrich 100–250 bp fragments was used. Samples were indexed during library preparation and sequenced in 20-plex using a Hiseq2500 (Illumina), 50 bp single-end reads.

For the semiconductor sequencing platform, shotgun libraries were prepared using a modified version of the Ion Plus fragment Library Kit (ThermoFisher Scientific) with end-repair using 2–10 ng of DNA. Size-selection was performed using a ratio of 0.8:1 beads to DNA solution with Agencourt AMpure beads (Beckman Coulter, Brea, CA). Ligation with Ion Xpress barcoded adapters in 50 µl was at 4 °C overnight followed by purification using Agencourt AMpure beads (Beckman Coulter, Brea, CA) and four or eight PCR cycles. NGS was performed using a Proton sequencing instrument after using an Ion Chef for template preparation as well as chip loading.

A 20-item Quality Management Plan (Supplementary Table S1) with predetermined quality thresholds was used for assessing the ongoing performance of NIPS assays in public laboratories during the testing phase.

Fetal fractions were estimated for each sample by SeqFF [13]. Fetal fraction estimates obtained from SeqFF were regressed on Y chromosomes-derived estimates from male pregnancies, used as reference, in order to check for the presence of bias. Chromosomal ratios were calculated for each sample as the number of reads of the chromosome of interest over the total number of reads from all autosomes after data processing by RAPIDR (v 0.1.1) [14].

The mean, standard deviation, and percent coefficient of variation (CV%) of each method for each chromosome of interest (chr 13, 18, 21, and X) were calculated from the results of a laboratory-and-method-specific reference set having between 17 and 130 euploid samples and tested in the same way as the other samples. The reference set was thus built from the results of known euploid samples. RapidR uses a binning process to clean the reference set by eliminating the outlier bins. The preserved bins, grouped by chromosome, are used for comparison between a sample and the reference set. Both NGS platforms results were analyzed with RapidR. The chromosome wide *z*-score from each study sample was then obtained. The cutoff for a positive NIPS result for any of the aneuploidies sought was a chromosome wide *z*-score of 3, based on published protocols as well as on preliminary verification of the sensitivity and specificity of the NIPS assays prior to finalization of assay protocols and testing of all patient samples. When the estimated fetal fraction (FF%) was below 4%, no result was reported (“NoCall”). NIPS assays were reported as “Failed” because of sample loss or unacceptable QC results (see Table S1) during the NIPS testing process.

To validate that these interpretation thresholds provide the best test accuracy we also calculated diagnostic sensitivity, specificity, and accuracy for other interpretation rules relative to reporting results with FF% from 0% to 4%, but also varying the *z*-score decision value between 2.5 and 5.

#### Reference standard

All fetuses received the reference standard. The reference standard was blind to index test result and undertaken using the existing clinical practice standards in the participating centers.

Thus, for all fetuses, the reference standard was either invasive testing with a fetal karyotype (or equivalent) for pregnancies having a positive screening result, or, for the remaining pregnancies, examination by a healthcare professional (pediatrician or family physician) at birth and a follow-up call at 6+ weeks of age. QF-PCR or microarray obtained after invasive testing were considered equivalent to the fetal karyotype with respect to their capacity to confirm or not the presence of a complete trisomy 13, 18, 21, or Turner syndrome in the fetus.

### Data management and statistical analyses

Data were collected in the central web-based relational PEGASUS database (MySQL). Statistical analyses were performed using SAS statistical package (v9.3). Clinical sensitivity, clinical specificity, PPV, NPV, and accuracy (ACC) were produced for each of the two index tests and the exact binomial confidence intervals calculated. Statistical tests comparing clinical sensitivity and specificity between the two index tests were conducted by using the Chi-Square or the Fisher’s exact tests for proportion. All tests were two-sided, testing the hypothesis that NIPS assays’ performance did not differ between index tests. McNemar–Bowker tests and paired *t*-tests were also performed to compare index tests. Alpha-error cutoff for significance was set at 0.01 given the many analyses performed.

Reference standard mosaic results or results involving chromosomes other than 13, 18, 21, or X were not considered as being among the aneuploidies that the screening program sought to detect and were therefore grouped with the normal karyotypes.

Index tests and the reference standard did not produce indeterminate test results except for missing data (or no/failed result). Missing data (i.e. no or failed result) on the index tests were not included in the performance analyses. Samples with a missing reference standard (*n* = 4) were not included in the analyses.

As pre-specified in the protocol, the variability in diagnostic accuracy was evaluated separately for high-risk pregnancies and pregnancies with baseline risk.

Sample size was estimated as follows. For an estimated sensitivity of 0.98 and specificity of 0.998 for NIPS assays, a 95% confidence interval width of 0.025 for sensitivity and 0.01 for specificity, a two side-alpha error of 0.05, the sample size for sensitivity was 3543 (121 cases with the disease), accounting for a prevalence of T21 cases of 1/30 in screen-positive women. For each study arm (high risk and baseline risk) a sample size of 1919 pregnancies provided 85% power (alpha error of 0.05; two-tailed test) to detect a 0.007 difference in specificity (after discussion with end users of the study) between NIPS methods for T13, T18, or T21, with an expected specificity of 0.998 and with a 95% confidence width for specificity of 0.002. We did not estimate sensitivity for T13, T18, or T21 in the baseline risk arm.

## Results

### Participating women and fetuses/infants

Figure 1 shows the participant and sample flow of the present study. Samples from a total of 3593 women were included in the final analysis. In addition to 329 women with euploid samples removed randomly (see above), 61 participants (1.7%) were lost to follow-up and 27 participants provided insufficient samples for NIPS analysis by both methods or failed to meet inclusion criteria upon review. Among 1933 high-risk women retained in the final analysis, based on invasive testing, there were 150 cases of Down syndrome (plus 1 mosaic), 49 cases of trisomy 18 (plus 3 mosaics), 8 cases of trisomy 13 (plus 2 mosaics), and 21 cases of Turner syndrome (45,X) (plus 1 mosaic). Among 1660 baseline risk women, based on the reference standard, there were five cases of Down syndrome and one case of Turner syndrome (plus one mosaic).

The characteristics of 3593 women included and the chromosomal status of the fetus or infant as determined by the reference standard are summarized in Table 1.

**Table 1:** Characteristics of women and chromosomal status of fetus/infant

Characteristics		High risk (*n* = 1933)	Baseline risk (*n* = 1660)
	*Age at edd*		
	Mean (median)	34.0 (34.0)	32.9 (33.0)
	SD (range)	5.4 (18–46)	4.5 (19–53)
	*Gestational age*		
	Mean (median)	17.1 (17.0)	12.2 (12.3)
	SD (range)	3.2 (10–23.9)	1.0 (10–13.9)
	*Weight (kg)*		
	Mean (median)	69.5 (65.8)	67.1 (63.5)
	SD (range)	14.9 (37–159)	15.0 (40–167)
	Missing	11	8
	*BMI, n (%)*		
	<18.5	33 (1.7)	74 (4.5)
	18.5–24.9	971 (50.9)	990 (60.2)
	≥25.0	902 (47.3)	582 (35.4)
	Missing	27	14
	*Ethnicity (%)*		
	Afro-Caribbean	29 (1.5)	39 (2.4)
	Asian	155 (8.1)	99 (6.0)
	European	1491 (77.6)	1272 (77.0)
	Oriental	184 (9.6)	188 (11.4)
	Other	62 (3.2)	54 (3.3)
	Missing	12	8
	*Gravidity (%)*		
	=1	482 (25.1)	636 (38.6)
	>1	1437 (74.9)	1012 (61.4)
	Missing	14	12
	*Parity (%)*		
	=0	751 (39.6)	933 (56.4)
	=1	786 (41.4)	553 (33.5)
	>1	360 (19.0)	167 (10.1)
	Missing	36	7
	*Smoking (%)*		
	No	1789 (92.8)	1600 (97.2)
	Yes	138 (7.2)	47 (2.9)
	Missing	6	13
	*Baby’s sex (%)*		
	Female	906 (46.9)	789 (47.5)
	Male	1027 (53.1)	871 (52.5)
	*Chromosomal status of fetus/infant (reference standard)*	(% of 1933–% of 2163)	(% of 1660–% of 1759)
	Euploid sample removed randomly	230	99
	Euploid sample tested	1651 (85.41)	1648 (99.28)
	Trisomy 13	8 (0.41–0.37)	0 (0–0)
	Trisomy 13—mosaic	2 (0.10–0.09)	0 (0–0)
	Trisomy 18	49 (2.53–2.27)	0 (0–0)
	Trisomy 18—mosaic	3 (0.16–0.14)	0 (0–0)
	Trisomy 21	150 (7.76–6.93)	5 (0.30–0.28)
	Trisomy 21—mosaic	1 (0.05–0.05)	0 (0–0)
	45,X	21 (1.09–0.97)	1 (0.06–0.06)
	45,X—mosaic	1 (0.05–0.05)	1 (0.06–0.06)
	47,XXX or 47,XXY	2 (0.10–0.09)	0 (0–0)
	Triploidy	10 (0.52–0.46)	3 (0.18–0.17)
	Abn other	35 (1.81–1.62)	2 (0.12–0.11)

### Test results

#### General results of index tests

The rate of samples not passing quality parameters (“fails”) was very small for both sequencing methods, 8/3593 for Proton™ sequencing and 1/3593 for HiSeq™ sequencing. This difference was not statistically significant (McNemar’s test, *p* value = 0.039). The mean number of filtered reads per sample was 6,728,475 for the Proton™ and 5,939,463 for the HiSeq™. The rate of “no call” (single and first assay) was 161/3593 for the Proton™ and 138/3593 for the HiSeq™ but this difference was not statistically significant (McNemar’s test, *p* value = 0.069).

The CV of chromosomal ratios is a critical parameter of NIPS test performance [15]. For euploid samples, the CV of the chromosomal ratio of the index method varied from 0.29 to 0.34 for chromosome 13, 0.31 to 0.38 for chromosome 18, 0.42 to 0.51 for chromosome 21, and 0.61 to 0.76 for chromosome X.

For aneuploid samples, the *z*-score ranged from 3 to 27 (Proton™) and 3.5 to 33 (HiSeq™) for T13, 3.4 to 30 (Proton™) and 3.4 to 37 (HiSeq™) for T18, 1.6 to 31.5 (Proton™) and 3 to 33 (HiSeq™) for T21 and −15 to −1.2 (Proton™) and −10 to 0 (HiSeq™) for 45,X.

**Fig. 2:**
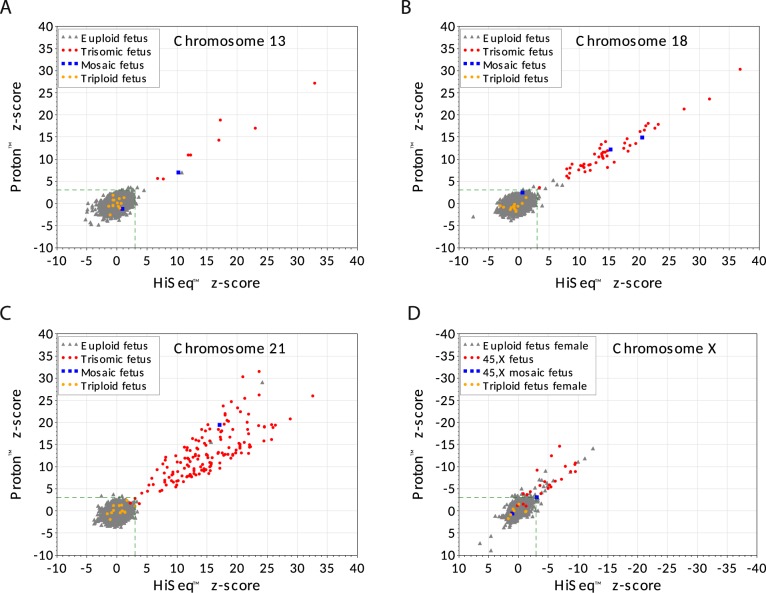
Individual *z*-scores and reference standard result for all fetuses tested with both index tests. Scatter plot of *z*-scores for same 3593 plasma samples comparing each index test (Proton (*Y*-axis) vs HiSeq (*X*-axis)) for chromosome 13, chromosome 18, chromosome 21, and chromosome X. Green triangles represent fetuses which, according to the reference standard, do not have the specific aneuploidy identified in the plot, while red circles represent fetuses that have the specific chromosome aneuploidy. Blue squares correspond to mosaic fetuses for the specific chromosome of interest, while yellow circles are triploid fetuses according to the reference standard. The lower left dashed green box in each plot shows the decision limit: *z*-score = 3

Figure 2 shows the *z*-scores for the same 3593 samples using the two index tests for trisomy 13, 18, 21, and 45,X. Both methods are in very high agreement for trisomies 13, 18, and 21 while more discrepancies and weaker clinical performances are observed for 45,X. Statistical analyses revealed that the two index tests were highly concordant for TP, FP, TN, and FN for each trisomy (Fisher’s exact test, all *p* value >0.73) and similar for 45,X with a *p* value of 0.045. For trisomies 13, 18, and 21 the *z*-score values were also correlated between the two index tests for positive samples (Pearson coefficients, T13 = 0.96, T18 = 0.96, T21 = 0.77, 45,X = 0.78, all *p* value ≤0.0001).

Fetal fraction as estimated by SeqFF varied between 0% and 35% and the difference between index tests was lower than 0.5% (means %FF, Hiseq™ = 11.0% and Proton™ = 11.1%, paired *t*-test *p* value <0.0001 for accepting the alternative hypothesis that Means %FF differed by less than 0.5%).

Among the high-risk samples with No call or a QCFail at the first test, there were 14.7% (11/75; Proton™ assay) cases of chromosomal anomalies, as compared to 12.3% (8/65) of HiSeq™ assay failures. As compared to samples with results, there was no statistically significant enrichment in fetal aneuploidy either in QCFails or NoCalls.

#### Concordance between results of index tests and reference method

Table 2 (upper half) presents the cross tabulation of each index test results (Proton™ and HiSeq™) by the results of the reference standard for the same 1933 high-risk pregnancies. Concordance between the two index methods is observed (Bowker’s test of symmetry with *p* values = 0.267 (Fail and No call grouped together).

**Table 2:** Concordance of tests results with reference standard, stratified for high-risk and baseline risk pregnancies

	Reference standard (fetus)												
	Normal	Trisomy 13		Trisomy 18		Trisomy 21		45,X		47,XXX 47,XXY	Triploidy	Abn other	Total
		Full	Mosaic	Full	Mosaic	Full	Mosaic	Full	Mosaic				
**High-risk pregnancies**													
*Proton™ results*													
Fail	4			1									5
No call	60	2			1	3		1			1	2	70
Normal	1561		1			2		3	1	2	9	31	1610
T13	5	6	1										12
T18	2			48	2							1	53
T21	3					145	1						149
Turner	16							17				1	34
Total	1651	8	2	49	3	150	1	21	1	2	10	35	1933
*HiSeq™ results*													
Fail	1												1
No call	56	1				3		1			1	2	64
Normal	1578		1		1	1		6	1	2	9	32	1631
T13	2	7	1										10
T18	2			49	2							1	54
T21	6					146	1						153
Turner	6							14					20
Total	1651	8	2	49	3	150	1	21	1	2	10	35	1933
**Baseline risk pregnancies**													
*Proton™ results*													
Fail	3												3
No call	89										2		91
Normal	1536										1	2	1539
T13	4												4
T18	3												3
T21	3					5							8
Turner	10							1	1				12
Total	1648	0	0	0	0	5	0	1	1	0	3	2	1660
*HiSeq™ results*													
Fail													0
No call	71										2	1	74
Normal	1565										1	1	1567
T13	4												4
T18	3												3
T21						5							5
Turner	5							1	1				7
Total	1648	0	0	0	0	5	0	1	1	0	3	2	1660

Cross tabulation of 1933 high risk (top rows) and 1660 baseline risk (bottom rows) pregnant women’s results by reference standard (columns) for the NIPS Proton™ (top half) and HiSeq™ (bottom half) assays. “Fail” samples correspond to samples for which quality thresholds for NIPS were not met, and “No call” samples correspond to samples for which the estimated fetal fraction was below 4% while the *z*-score was too low. Data boxes with zero value are left empty to facilitate reading

Table 2 (lower half) shows the cross tabulation of each index test results by the reference standard for the same 1660 baseline risk pregnancies. Concordance between the two index tests is also observed in this category of pregnancies (Bowker’s test of symmetry with *p* values = 0.812; Fail and No call grouped together).

When comparing results on the index tests and reference method for samples that generated a result for both the Proton™ and HiSeq™ assays, discordant results were seen for 66 cases in which the fetus was normal according to the reference method (i.e. there were false-positive results for one or both assays) and 8 aneuploid cases (false-negative results for one or both assays). Of the 66 false-positive cases, Proton™ and HiSeq™ results were concordant in 19 cases (4 cases of T13, 6 cases of T18, 3 cases of T21, and 6 cases of 45,X). Five of those 19 cases could be explained by fetal mosaicism confirmed by the reference standard and classified as per protocol as “normal” (1 mosaic T13, 2 mosaic T18, 1 mosaic T21, and one 45,X mosaic). Of the eight false-negative cases, four had concordant index tests results (one false-negative T21 and three false-negative 45,X with both index tests).

### Diagnostic accuracy and predictive value estimates

Table 3 shows the 2 × 2 tables and diagnostic accuracy estimates for each index test in the 1933 high-risk pregnancies. Clinical sensitivity, clinical specificity, negative predictive value (NPV), false positive rates, and the accuracy are excellent for trisomy 21, 18, and 13 but for trisomy 13 confidence intervals of clinical sensitivity are wider due to a small number of cases. For T13, T18, and T21, the results are very similar with very strong specificity estimates (≥99.5%) and NPVs (≥99.8%) (Chi-square or Fisher’s exact test with *p* values between 0.342 and 1.000). Both index tests show weaker and similar performance for detecting Turner syndrome with a sensitivity of 85% for Proton™ and 70% for HiSeq™ (Chi-square *p* value = 0.09), and a specificity of 99% for Proton™ and 99.6% for HiSeq™(Chi-square *p* value = 0.021).

**Table 3:** Diagnostic performance of index tests, stratified for high risk and baseline risk pregnancies

Fetal anomaly	TP	FP	TN	FN	FPR Pr (FP|N)	SPEC Pr (−|N)	SENS Pr (+|A)	PPV Pr (A|+)	NPV Pr (N|−)	ACC Pr (TP+TN|all)
	*n*	*n*	*n*	*n*	*n*, % (95% CI)	*n,* % (95% CI)	*n,* % (95% CI)	*n,* % (95% CI)	*n,* % (95% CI)	*n,* % (95% CI)
**High-risk pregnancies**										
*Proton™*										
T21	145	4	1707	2	4/1711	1707/1711	145/147	145/149	1707/1709	1852/1858
					0.23 (0.06–0.60)	99.7 (99–100)	98.6 (95–100)	97.3 (93–100)	99.8 (99–100)	99.6 (99–100)
T18	48	5	1805	0	5/1810	1805/1810	48/48	48/53	1805/1805	1853/1858
					0.28 (0.09–0.64)	99.7 (99–100)	100 (92–100)	90.5 (79–97)	100 (99–100)	99.7 (99–100)
T13	6	6	1846	0	6/1852	1846/1852	6/6	6/12	1846/1846	1852/1858
					0.32 (0.12–0.70)	99.6 (99–100)	100 (54–100)	50.0 (21–79)	100 (99–100)	99.6 (99–100)
Turner	17	17	1821	3	17/1838	1821/1838	17/20	17/34	1821/1824	1838/1858
					0.92 (0.54–2)	99.0 (98–100)	85.0 (62–97)	50.0 (32–68)	99.8 (99–100)	98.9 (98–100)
T13, T18, or T21	199	15	1642	2	15/1657	1642/1657	199/201	199/214	1642/1644	1841/1858
					0.91 (0.51–2)	99.0 (98–100)	99.0 (96–100)	92.9 (88–97)	99.8 (99–100)	99.0 (98–100)
*HiSeq™*										
T21	146	7	1714	1	7/1721	1714/1721	146/147	146/153	1714/1715	1860/1868
					0.41 (0.16–0.84)	99.5 (99–100)	99.3 (96–100)	95.4 (90–99)	99.9 (99–100)	99.5 (99–100)
T18	49	5	1814	0	5/1819	1814/1819	49/49	49/54	1814/1814	1863/1868
					0.27 (0.09–0.64)	99.7 (99–100)	100 (92–100)	90.7 (79–97)	100 (99–100)	99.7 (99–100)
T13	7	3	1858	0	3/1861	1858/1861	7/7	7/10	1858/1858	1865/1868
					0.16 (0.03–0.47)	99.8 (99–100)	100 (59–100)	70.0 (34–94)	100 (99–100)	99.8 (99–100)
Turner	14	6	1842	6	6/1848	1842/1848	14/20	14/20	1842/1848	1856/1868
					0.32 (0.12–0.71)	99.6 (99–100)	70.0 (45–89)	70.0 (45–89)	99.6 (99–100)	99.3 (98–100)
T13, T18, or T21	202	15	1650	1	15/1665	1650/1665	202/203	202/217	1650/1651	1852/1868
					0.90 (0.51–2)	99.0 (98–100)	99.5 (97–100)	93.0 (88–97)	99.9 (99–100)	99.1 (98–100)
**Baseline risk pregnancies**										
*Proton™*										
T21	5	3	1558	0	3/1561	1558/1561	—	—	—	—
					0.19 (0.04–0.56)	99.8 (99–100)	—	—	—	—
T18	0	3	1563	0	3/1566	1563/1566	—	—	—	—
					0.19 (0.04–0.56)	99.8 (99–100)	—	—	—	—
T13	0	4	1562	0	4/1566	1562/1566	—	—	—	—
					0.26 (0.07–0.65)	99.7 (99–100)	—	—	—	—
Turner	1	11	1554	0	11/1565	1554/1565	—	—	—	—
					0.70 (0.35–2)	99.2 (98–100)	—	—	—	—
T13, T18 or T21	5	10	1551	0	10/1561	1551/1561	—	—	—	—
					0.64 (0.31–2)	99.3 (98–100)	—	—	—	—
*HiSeq™*										
T21	5	0	1581	0	0/1581	1581/1581	—	—	—	—
					0 (0–0.23)	100 (99–100)	—	—	—	—
T18	0	3	1583	0	3/1586	1583/1586	—	—	—	—
					0.19 (0.04–0.55)	99.8 (99–100)	—	—	—	—
T13	0	4	1582	0	4/1586	1582/1586	—	—	—	—
					0.25 (0.07–0.64)	99.7 (99–100)	—	—	—	—
Turner	1	6	1579	0	6/1585	1579/1585	—	—	—	—
					0.38 (0.14–0.82)	99.6 (99–100)	—	—	—	—
T13, T18, or T21	5	7	1574	0	7/1581	1574/1581	—	—	—	—
					0.44 (0.18–0.91)	99.5 (99–100)	—	—	—	—

Diagnostic performance of the two index tests (Proton™ NIPS and HiSeq™ NIPS) for T21, T18, T13; Turner syndrome and the detection of any of T13, T18, or T21 (rows) in high-risk pregnancies (top half) and baseline risk pregnancies (bottom half). The first four data columns represent the 2 × 2 table absolute frequencies observed as true positives (TP), false positives (FP), true negatives (TN), and false negative (FN), followed, for high-risk pregnancies, with the false positive rate (FPR), the clinical sensitivity (SENS), the clinical specificity (SPEC), the positive predictive value (PPV), the negative predictive value (NPV), and the clinical accuracy (ACC) while, for baseline risk pregnancies, only are shown the false positive rate (FPR) and the clinical specificity (SPEC). For each ratio, the corresponding table box contains the absolute ratio (*n*), the relative ratio (%), and the 95% confidence interval of the relative ratio (95% CI)

When we consider the performance of index tests for the detection of any of T13, T18, or T21, it appears that both index tests have a clinical sensitivity of ≥99% (95% CI 96—100%), a clinical specificity of 99% (95% CI 98—100%), an NPV of ≥99.8% (95% CI 99—100%), a combined FPR (T13, T18, or T21) of 0.9% (95% CI 0.5—2%) and an ACC ≥99% (lower limit of 95% CI of 98%). The PPV is 93% (lower limit of 95% CI is 88%).

Table 3 (bottom half) shows the 2 × 2 tables and diagnostic accuracy estimates for each index test in the 1660 baseline risk pregnancies. Given the very small number of cases of aneuploidy in this group, we present only the FPR and clinical specificity estimates which are similar for both index tests as seen from the largely overlapping confidence intervals for all estimates.

With respect to fetal sex determination, the Proton™ NIPS assay correctly identified 99.7% (1847/1852) male fetuses (47 NoCalls) and 98.9% (1647/1665) female fetuses (29 NoCalls), while the HiSeq™ NIPS assay identified respectively 99.7% (1878/1883) male (16 NoCalls) and 99.2% (1665/1678) female fetuses (16 NoCalls). Overall, these differences were not statistically significant for males (Chi-square *p* value = 0.98) or females (Chi-square *p* value = 0.36).

### Comparative diagnostic accuracy and predictive values

Comparison of both index tests (Table 4), which was the major objective of this study, revealed that there was no overall statistically significant difference between index tests for clinical sensitivity, clinical specificity, PPV, NPV, false positive rate nor accuracy, either for T21, T18, T13 or 45,X.

**Table 4:** Comparison of diagnostic performance of index tests—any risk

		SENS	SPEC	PPV	NPV	FPR	ACC
	**T13**						
	Proton™	6/6	3408/3418	6/16	3408/3408	10/3418	3414/3424
	HiSeq™	7/7	3440/3447	7/14	3440/3440	7/3447	3447/3454
	*p* value	—	0.456	0.491	—	0.456	0.455
	**T18**						
	Proton™	48/48	3368/3376	48/56	3368/3368	8/3376	3416/3424
	HiSeq™	49/49	3397/3405	49/57	3397/3397	8/3405	3446/3454
	*p* value	—	0.986	0.970	—	0.986	0.986
	**T21**						
	Proton™	150/152	3265/3272	150/157	3265/3267	7/3272	3415/3424
	HiSeq™	151/152	3295/3302	151/158	3295/3296	7/3302	3446/3454
	*p* value	1.000	0.986	0.990	0.623	0.986	0.794
	**45,X**						
	Proton™	18/21	3375/3403	18/46	3375/3378	28/3403	3393/3424
	HiSeq™	15/21	3421/3433	15/27	3421/3427	12/3433	3436/3454
	*p value*	0.454	0.0103	0.173	0.508	0.0103	0.058
	**Any of T13, T18, or T21**						
	Proton™	204/206	3193/3218	204/229	3193/3195	25/3218	3397/3424
	HiSeq™	207/208	3224/3246	207/229	3224/3225	22/3246	3431/3454
	*p* value	0.622	0.639	0.644	0. 623	0.639	0.549

Comparison of the two index tests in all samples tested (any risk) for fetal T13, T18, T21, Turner syndrome and any of T13, T18, or T21 (rows). For each fetal anomaly index tests are compared for clinical sensitivity (SENS), clinical specificity (SPEC), positive predictive value (PPV), negative predictive value (NPV), false positive rate (FPR), and clinical accuracy (ACC). Absolute ratios are presented with the *p* value of the Chi-Square or Fisher’s exact test statistics, depending on the number of results available

### Impact of varying FF% and *z*-score decision values on diagnostic accuracy and NoCall rates

We investigated the potential impact on diagnostic accuracy of reporting results with FF% between 0% and 4%. We also analyzed the consequences on diagnostic accuracy of raising the *z*-score cutoff between 2.5 and 5 for reporting a high risk of aneuploidy. The 2 × 2 results for each of these 30 FF%–*z*-score threshold combinations are shown, for high-risk pregnancies, in Supplementary Tables S2 for T13, T18, T21, 45,X, and any of T13, T18 or T21.

In high-risk pregnancies, reporting all results using a *z*-score threshold of 3.0 and without taking FF% into account would have yielded, for the Proton™ assay, an additional four true positives (two T13 and two T21), 3 false positives, 62 true negatives, and 1 false-negative result (missing one T21 case). For the HiSeq™ assay the same thresholds would have yielded an additional four true positives (one T13 and three T21), two false positives, 58 true negatives, and no false-negative result. These differences are not significant (Fisher exact test, *p* value = 0.85).

As expected, reporting results for samples with lower FF% significantly improves the NoCall rate of NIPS assays and brings it, for instance in high-risk pregnancies, from 3.9% (Proton™) and 3.4%(HiSeq™) at a 4% FF cutoff, to 2% and 1.76% at a 3% FF cutoff to 1% and 0.9% at a 2% FF cutoff, and to 0.6% and 0.3% at a 1% FF cutoff, while still having few false-negative cases.

## Discussion and conclusion

While, in many jurisdictions, NIPS is becoming the standard of care as a second-tier screening test for fetal aneuploidy, studies about the clinical performance of NIPS have been affected by many biases [3, 4, 16]. It was one of our objectives to compare head-to-head (using the exact same samples) the diagnostic accuracy of two very different sequencing technologies such as to increase the technological options for implementing NIPS in clinical laboratories worldwide.

### General interpretation of results

The PEGASUS prospective and independent study clearly shows that different platforms of massively parallel shotgun sequencing of unselected ccfDNA fragments from maternal blood are very sensitive. In high-risk pregnancies, both index tests showed a clinical sensitivity of 98.6% or more for T21, T18, and T13 and their PPV was of 95% for T21, 90% for T18, but of 70% or less for T13 and 45,X. Both index tests also appeared highly specific (i.e. clinical specificity of 99.5% with in high-risk pregnancies an NPV of 99.8% for T13, T18, T21, and 99.6% for 45,X) methods for the detection of fetal trisomies 13, 18, and 21.

This study was appropriately powered to detect clinically significant test performance differences between the two index tests for T21 and T18, and we observed no statistically significant difference in sensitivity, specificity, PPV, or NPV for these two conditions. We observed no statistically significant difference for 45,X and T13; however, the number of cases was insufficient to detect differences in test sensitivity for these conditions. Both index tests showed no difference in their chromosomal ratio coefficients of variation for chromosomes 13, 18, 21, X, range of fetal fraction estimates, and range of *z*-scores for each of these different chromosomal imbalances. No statistically significant difference was observed between index tests for either the Fail rate or the NoCall rate.

In baseline risk pregnancies, both index tests showed similar clinical specificity as in higher risk pregnancies. Given the low frequency of fetal aneuploidy in baseline risk pregnancies (combined T21, T18, and T13 estimated at 0.31% of first trimester pregnancies), we can predict from the observed FPR of 0.44% that the PPV of NIPS for any of T13, T18, or T21 should be about 40%. The NPV will necessarily be high in baseline risk pregnancies given the already low a priori frequency of fetal aneuploidy before testing.

We observed a high degree of concordance between the two index tests, which used different sequencing platforms and were performed in different physical laboratories. This suggests that, even if these were both laboratory developed NIPS assays, provided they are performed by experienced clinical laboratories, using a highly detailed quality management plan and well validated analytically, they can provide equivalent clinical test performance. This finding may be very useful for smaller laboratories that would not expect to receive enough samples per week to launch a NIPS procedure on a very high-throughput sequencing instrument. Although it has been previously reported, based on results from 2275 pregnant women, that semiconductor sequencing (Ion Proton˘) was appropriate for clinical diagnostic laboratories, the two NGS platforms were not compared head-to-head [11]. To our knowledge, the only such report is one comparing the performance of MiSeq™ (Illumina) and Ion Proton™ platforms for 18 cases of spontaneous abortion of different aneuploidies. The authors found a similar performance for these two platforms [17] but did not have the statistical power to detect clinically relevant differences in performance.

In the present study, we observed 23 cases where both index tests results were in disagreement with the reference standard (19 false-positive and 4 false-negative results). There were such cases for T21 (three false positives, one false negative), for T18 (six false positive), for T13 (four false positives), and for 45,X (three false positives and six false negatives). These were not consecutive samples nor samples taken at the same locations in the same days, thus unlikely to be sample swaps. Five of these false positive (one T13, two T18, one T21, and one 45,X) are explained by confirmed fetal mosaicism for the chromosomal abnormality detected. These cases as per our methodology were grouped with the normal as the aim of screening was to detect full trisomies and monosomy X. The other false positives with both index tests are likely due to mosaicism confined to the placenta or maternal mosaicism rather than to analytical errors [18]. However, one limitation of the present study is the lack of placental material to determine the proportion of FP and FN that could be due to mosaicism confined to the placenta.

To our knowledge, no diagnostic test accuracy study on NIPS published up to now has performed threshold analysis to determine the most efficient decision cut-offs for reporting results in terms of FF% and for reporting high-risk pregnancies in terms of chromosomal *z*-score. Our threshold analysis suggests that test accuracy may possibly be increased by using a *z*-score cutoff different than 3. Given the strong discrimination power of NIPS observed in the present study, it may appear worthwhile to consider raising the *z*-score cutoff pending studies with more cases in this range of *z*-scores. Our analysis of the small number of pregnancies with FF% below 4% (including only a handful of cases of fetal aneuploidy) suggests that, pending further studies, it may be interesting to consider reporting results with low FF% as this would significantly lower the NoCall rate while still not missing many cases. Further studies with more samples with %FF below 4% will be needed to confirm these findings.

### Study limitations, sources of potential bias, statistical uncertainty, generalizability

The present study was conceived with a prospective design for measuring and comparing the diagnostic test accuracy of two different NIPS sequencing methods.

Recruitment occurred in five different sites across Canada. Therefore, the characteristics of recruited women may not be representative of the whole pregnant women population of Canada, limiting generalizability. However, given that NIPS performance appears to be similar in different countries [3], we do not believe that this lack of generalizability strongly affects our conclusions. In addition, for high-risk pregnancies, the number of referral centers is limited in each province in Canada and we included many of those centers in PEGASUS. Furthermore, the study design aimed at minimizing many other potential sources of bias. The PEGASUS study protocol and sample size were planned before initiation of the trial, participants were recruited prospectively, samples were randomized between laboratories prior to performing the index tests, they were further randomized within each lab to determine their testing order. Further, NIPS assays were performed as well as their results reported blind to the study arm, pregnancy risk of aneuploidy, maternal variables, pregnancy and fetal outcomes, recruitment site, and laboratory which purified the plasma before freezing.

In terms of method performance, due to the study design, PEGASUS laboratories performed NIPS assays on plasma samples that had been frozen after the high-speed centrifugation step and before extracting ccfDNA, as opposed to having processed all samples from sampling to sequencing without freezing. Thus, the observed fail rate of NIPS assays studied may not be the same as for NIPS using fresh samples. However, our results show that freezing plasma before ccfDNA extraction does not seem to affect significantly the NIPS clinical performances when results can be provided. Also, our results show that samples from baseline pregnancies that failed the first attempt for an NIPS assay had a risk of 2–4% (depending on the assay) of having a fetus with a chromosomal anomaly other than T13, T18, or T21 (reference standard). This proportion was 12–15% in high-risk pregnancies samples that failed the first attempt at NIPS. However, amongst failed NIPS first attempts in high-risk pregnancies, we observed no statistically significant enrichment of cases of T18 and T21 and 45,X (absolute proportions were lower than euploids or *p* value > 0.240). The proportion of T13 showed a trend to be slightly higher in no-result than in euploids (2/8 vs 64/1651, *p* value = 0.0374) for the Proton™ than the HiSeq™ (1/8 vs 56/1651, *p* value = 0.244). Our results suggest that in clinical practice, high-risk women with a failed NIPS result should be offered invasive diagnostic testing given their a priori increased risk for a chromosomal abnormality and their later gestational age. For the population of women with a baseline risk and a failed NIPS result, our study does not indicate that such a result indicates an increased risk of trisomy 21, 18, or 13 but rather an increased risk of other severe chromosomal anomalies such as triploidy. For these women, ultrasound examination should be initiated, followed by counseling regarding the options of either repeating the NIPS, pursuing another form of screening (serum biochemistry and NT ultrasound) or proceeding with invasive diagnostic testing depending on results of the ultrasound examination.

With respect to statistical uncertainty, the present study was powered to compare the clinical performance of the index tests in high-risk pregnancies. For pregnancies with baseline risk, this study was powered only to compare clinical specificity and NPP between methods. Indeed, due to the low prevalence of these aneuploidies in such pregnancies, about 100,000 pregnancies would need to be recruited in order to obtain adequate power for all diagnostic performance parameters. This limitation has affected all of the small number of studies that have investigated NIPS performance in pregnancies with baseline risk [3]. Our sample size of 1660 provided 85% power to detect a difference of 0.008 in specificity (vs 0.998 specificity) with an alpha error of 0.05. Altough we performed multiple comparisons in the present paper, we chose, as advised by Perneger [19], to describe what test of significance have been performed rather than making a correction for multiple comparisons given that alpha-error corrections are at the expense of power and the present study hypothesis was the equivalence of index tests.

It appears clear from our study that, for the detection of any of the three main trisomies (T13, T18, and T21), the two index tests do not differ (upper 99% confidence interval limit of the difference) by more than 2.6% in clinical sensitivity, 0.64% in clinical specificity, 0.64% in FPR, and 0.65% in ACC. In terms of PPV and NPV, in high-risk pregnancies, index tests differed by less than 6.41% for PPV and 0.33% for NPV. In pregnancies with baseline risk only the NPV could be reliably estimated and it did not differ (upper 99% confidence interval limit of the difference) by more than 0.33% between the two index tests. As we randomly removed 99 baseline risk and 230 high-risk euploid samples from the NIPS testing processes, we estimate that we may have missed only two potential false-positive results, which would lower the overall PPV estimates by about 0.6%, which is not clinically significant.

In terms of generalizability of findings, the present study faces similar limitations as for any such study. It was realized in Canada, mainly in academic centers, index tests were performed in a limited number of laboratories that were well versed in molecular diagnostics, as well as genetic and genomic clinical tests validation [20–23]. However, the fetal aneuploidies studied have the same prevalence in most countries, and NIPS has not been shown to be influenced by the genetic background of pregnant women. Thus, we believe that our findings should be generalizable to other populations, conditional on each NIPS clinical laboratory performing a thorough analytical validation of their laboratory developed NIPS assay as recommended by best practice guidelines for NGS methods [24, 25]

### Implications for practice including intended use of index test

Most clinical guidelines for prenatal screening of fetal aneuploidy recommend that NIPS be used as a second-tier screening test to remove the many false-positive results generated by traditional screening schemes (biochemical with or without ultrasound). Our results confirm the excellent clinical performance of genomics-based non-invasive prenatal testing using ccfDNA in maternal blood. The very high estimates of the ACC (lower estimate of the 95% IC ≥ 99.5% for T13, T18, or T21) of NIPS performed with either optical-based MPSS NIPS or semiconductor-based MPSS NIPS underscore the high clinical sensitivity and specificity of both index tests.

The great similarity between index tests results for all estimates of performance parameters also suggests that they are undistinguishable and will provide equivalent quality in their results. NGS-based whole-genome approaches are thus a very strong and reliable platform as the results are reliable even with large differences in approaches. This opens the possibility for clinical laboratories that wish to implement NIPS assays to choose between these two sequencing platforms according to their needs in terms of infrastructure funds, test throughput, turnaround time, batch sizes, and costs. In sum, in the present study IonTorrent™ (ThermoFisher) sequencing on the Proton™ was usually cheaper, enabled samples to be analyzed smaller batches, and was more rapid than sequencing by sequence ™ (Illumina) on HiSeq2500™ instruments. However, NGS sequencing platforms evolve rapidly and these differences may not hold with newer generations of NGS equipment and technologies such as, for instance, the midsize NextSeq™ instruments from Illumina which enable analysis of small batches and is more rapid.

Importantly, the PPV estimates of both index tests however confirmed again that NIPS should not be considered as a diagnostic test and that all positive results should be followed by a confirmatory diagnostic (invasive) test before any irreversible decision is made by pregnant women and their partner.

Future research avenues that will need to be tackled include obtaining better estimates of the clinical sensitivity, FPR, and PPV of first trimester NIPS, further studies into the causes of FP and FN results, better powered studies about the impact on diagnostic accuracy of reporting results below an FF% of 4%, and larger studies to address the issues we have raised about optimizing the *z*-score decisional thresholds.

## Supplementary information

Supplementary materials S1, S2a-S2e
