# Supplementary material for: Prospective head-to-head comparison of accuracy of two sequencing platforms for screening for fetal aneuploidy by cell-free DNA: the PEGASUS study
Source: Eur J Hum Genet. 2019 Jun 23;27(11):1701–15. doi: 10.1038/s41431-019-0443-0 (PMC6871532; doi:10.1038/s41431-019-0443-0)
Supplement: Supplementary file 1 — Supplementary materials S1, S2a-S2e [file 41431_2019_443_MOESM1_ESM.docx]

Supplementary Materials

Supplementary Table S1–Quality Criteria and Thresholds of Index tests

| **QC Domain** | **Item** | Criterion | Comments |
| --- | --- | --- | --- |
| **1-Sample qualification and storage (quantity/quality)** |  |  |  |
| sample characteristics | 1.1 sample plasma volume | 3-4 ml | In one lab, 2.5 ml was needed. |
|  |  |  |  |
|  | 1.2 temperature of sample upon reception | Room temperature or above 4°C |  |
|  |  |  |  |
| delay between blood draw and plasma separation | 1.3 Streck tube | 5 days |  |
|  |  |  |  |
|  | 1.4 EDTA tube | 24 h |  |
|  |  |  |  |
| **2-Instrument and environment qualification** |  |  |  |
| instrument | 2.1 log of parameters vs specifications | yearly |  |
|  |  |  |  |
|  | 2.2 log of instrument maintenance and repair | according to events |  |
|  |  |  |  |
| environment | 2.3 log of lab ambient temperature and humidity | na | In one lab, temperature and humidity is monitored in real-time; max/min thresholds will alarm Temp 28/13 (~21=set point); Humidity 65/25 (40=set point) Eschelon Building. |
|  |  |  |  |
| **3-ccfDNA qualification** |  |  |  |
|  | 3. 1 total DNA recovery | at least 3 ng |  |
|  |  |  |  |
|  |  |  |  |
| **4-Library qualification** | Item | Thresholds | |
|  |  | HiSeq™ | Proton™ |
| quantity (pmol) | 4.1 concentration (pmol) | 2nM | >300 pmol |
|  |  |  |  |
| quality | 4.2 Size according to Bioanalyser profile | 305 to 350bp | Bioanalyser only if qPCR>Qubit value which may means presence of concatemers |
|  |  |  |  |
| **5-Sequencing qualification** |  | HiSeq™ | Proton™ |
|  | 5.1 % loading | 70% Q30/85% Q20/>70% Passing filter | > 80% |
|  |  |  |  |
|  | 5.2 nb of usable reads | Rapid Mode: Total 370 M reads/2 lanes | > 60 million per P1 chip |
|  |  |  |  |
|  | 5.3 mean read length | 50bp SE reads | Between 140 and 160 bp |
|  |  |  |  |
|  |  |  |  |
|  | 5.4 Nb raw reads per patient sample (library) | At least 5M | At least 5M and pool of patients with equivalent number of reads per sample. |
|  |  |  |  |
|  | 5.5 Raw cluster per lane/well with live ISP | 800 to 1050K/mm^2^ | > 85% |
| **6-Analysis qualification and NIPT scores** |  | HiSeq™ | Proton™ |
|  | 6.1 Number of reads remaining after filtering | > 4M | > 4M |
|  |  |  |  |
|  | 6.2 Contamination deduced from spiked DNA | Less than 1% for one lab and less than 5% for the other one. | Less than 1% |
|  |  |  |  |
|  | 6.3 One control aneuploid per chip | Control must be called aneuploid (Westgard rule 1_2s_) | Control must be called aneuploid for control chromosome (Westgard rule 1_2s_) but euploid for all others and right sex |
|  |  |  |  |
|  |  |  |  |
|  | 6.4 Run variation | One lab used normalization to adjust for inter-run variability. | One lab used normalization to adjust for inter-run variability. |
|  |  |  |  |
|  | 6.5 Fetal fraction | >= 4% | >= 4% |
|  |  |  |  |

Supplementary Table S2a – Threshold analysis for trisomy 13 in high-risk pregnancies

Table S2a Legend – Threshold analysis for the detection of trisomy 13 in high-risk pregnancies. The table presents, for each index test (left half and right half of the table), the number of samples with No Calls, true positive, false positive, true negative, and false negative results, the estimated clinical sensitivity, clinical specificity and clinical accuracy in %, for different combinations (rows) of fetal fraction reporting thresholds and z-scores for a positive screening result. The green line represents the cut-offs used for the main results presented (4%FF and z-score of 3), while the yellow boxes show the thresholds providing the highest accuracy.

Supplementary Table S2b – Threshold analysis for trisomy 18 in high-risk pregnancies

Table S2a Legend – Threshold analysis for the detection of trisomy 18 in high-risk pregnancies. The table presents, for each index test (left half and right half of the table), the number of samples with No Calls, true positive, false positive, true negative, and false negative results, the estimated clinical sensitivity, clinical specificity and clinical accuracy in %, for different combinations (rows) of fetal fraction reporting thresholds and z-scores for a positive screening result. The green line represents the cut-offs used for the main results presented (4%FF and z-score of 3), while the yellow boxes show the thresholds providing the highest accuracy.

Supplementary Table S2c– Threshold analysis for trisomy 21 in high-risk pregnancies

Table S2a Legend – Threshold analysis for the detection of trisomy 21 in high-risk pregnancies. The table presents, for each index test (left half and right half of the table), the number of samples with No Calls, true positive, false positive, true negative, and false negative results, the estimated clinical sensitivity, clinical specificity and clinical accuracy in %, for different combinations (rows) of fetal fraction reporting thresholds and z-scores for a positive screening result. The green line represents the cut-offs used for the main results presented (4%FF and z-score of 3), while the yellow boxes show the thresholds providing the highest accuracy.

Supplementary Table S2d– Threshold analysis for 45, X in high-risk pregnancies

Table S2a Legend – Threshold analysis for the detection of 45, X in high-risk pregnancies. The table presents, for each index test (left half and right half of the table), the number of samples with No Calls, true positive, false positive, true negative, and false negative results, the estimated clinical sensitivity, clinical specificity and clinical accuracy in %, for different combinations (rows) of fetal fraction reporting thresholds and z-scores for a positive screening result. The green line represents the cut-offs used for the main results presented (4%FF and z-score of 3), while the yellow boxes show the thresholds providing the highest accuracy.

Supplementary Table S2e– Threshold analysis for any of T13, T18 or T21 in high-risk pregnancies

Table S2a Legend – Threshold analysis for the detection of any of T13, T18 or T21 in high-risk pregnancies. The table presents, for each index test (left half and right half of the table), the number of samples with No Calls, true positive, false positive, true negative, and false negative results, the estimated clinical sensitivity, clinical specificity and clinical accuracy in %, for different combinations (rows) of fetal fraction reporting thresholds and z-scores for a positive screening result. The green line represents the cut-offs used for the main results presented (4%FF and z-score of 3), while the yellow boxes show the thresholds providing the highest accuracy.
